# Opacités nodulaires diffuses et calcifiées

**DOI:** 10.11604/pamj.2016.24.205.9169

**Published:** 2016-07-08

**Authors:** Hasna Jabri, Régis Gothard Bopaka, Nawal Lakhdar, Houda Moubachir, Wiam El Khattabi, Hicham Afif

**Affiliations:** 1 Service des Maladies Respiratoires, Hôpital 20 Août 1953, CHU Ibn Rochd, Casablanca

**Keywords:** Opacité nodulaire, biopsie, adénocarcinome, Nodular opacity, biopsy, adenocarcinoma

## Abstract

L’adénocarcinome pulmonaire est difficile à évoquer d’emblée devant les données anamnestiques et même radiologiques. Nous rapportons une observation d’une femme présentant une dyspnée avec la radiographie une opacité micronodulaire disséminée, confluente dans les deux champs pulmonaires avec des calcifications par endroit. L’histologie à travers les biopsies transbronchiques a permis de poser le diagnostic. Le pronostic était sombre par le décès de la patiente.

## Abstract (en)

Pulmonary adenocarcinoma is difficult to identify right away with respect to anamnestic and even to radiological data. We here report the case of a woman with dyspnea. Radiological examination showed disseminated micronodular opacity confluent in both lung fields with calcifications in certain locations. Histological examination of transbronchial biopsies allowed the diagnosis. The prognosis was poor with the death of the patient.

## Introduction

L’adénocarcinome pulmonaire est une tumeur maligne de différentiation glandulaire développée au niveau du parenchyme pulmonaire. Elle est fréquente et de pronostic sombre. Son incidence est en augmentation chez la femme. Le diagnostic repose sur les l’anamnèse, la clinique, l’imagerie et la preuve histologique. Nous rapportons une observation d’une femme présentant une dyspnée avec une opacité micronodulaire disséminée, confluente dans les deux champs pulmonaires à la radiographie thoracique. Chez qui le diagnostic de l’adénocarcinome pulmonaire n’était pas aisé.

## Patient et observation

**Figure 1:**
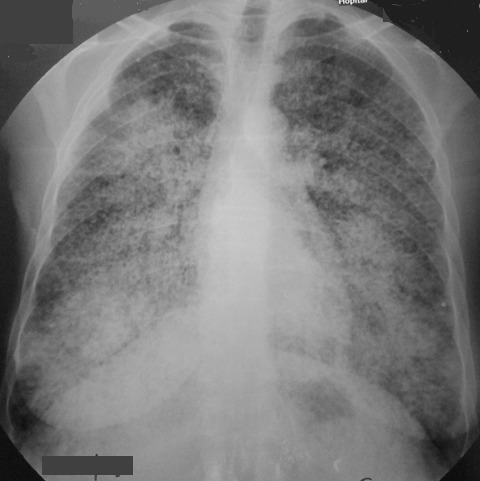
Radiographie thoracique de face: opacité micronodulaire disséminée, confluente dans les deux champs pulmonaires

**Figure 2:**
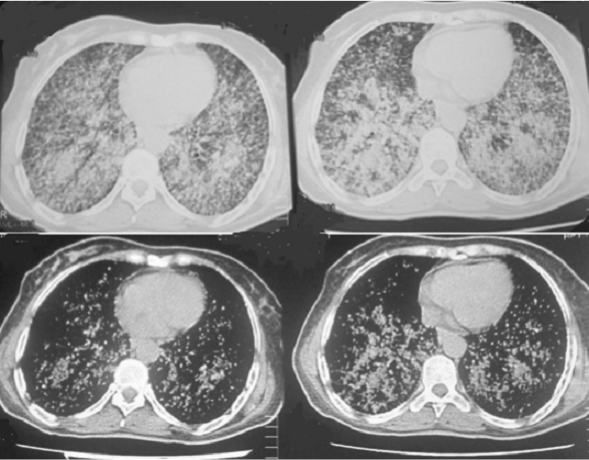
Tomodensitométrie thoracique avec injection de produit de contraste en coupes parenchymateuses millimétrées: micronodules associées à des nodules diffus, calcifiés dans certains endroits

Une femme âgée de 68 ans, dyspnéique depuis 3 mois sans autres signes respiratoires ni extra-respiratoires, suivie depuis 30 ans pour asthme contrôlé sous l’association fluticasone/formotérol 1000μg/j, et pour diabète type 2 depuis 14 ans sous glyclazide. Elle n’a pas d’antécédents toxiques, notamment tabagiques. A l’admission, la patiente présentait une polypnée à 28 cycles/min, une saturation percutanée à 93% sous oxygène, une pression artérielle à 120/80 mmHg, une fréquence cardiaque à 80 batt/min et une température à 37°C. L’examen clinique notait des râles crépitants dans les deux champs pulmonaires prédominants aux deux bases. Le reste de l’examen clinique était sans particularité. Les gaz du sang à l’air ambiant montraient un pH à 7,45, une PaO2 à 45,9 mmHg, une PaCO2 à 31,4 mmHg et des bicarbonates à 21,7 mmol/l. L’examen biologique montrait une glycémie à jeun de 2,61g/l à deux reprises avec une hémoglobine glyquée à 8,10%. La numération formule sanguine montrait une hyperleucocytose à 14510/mm3 à prédominance polynucléaire à 8360/mm3 avec une hyperéosinophilie à 1630/mm3. La vitesse de sédimentation était à 25mmà la première heure. L’électrophorèse de protéine montrait une hypergammaglobulinémiepolyclonale modérée à 15,84g/l. La créatininémie, l’urée, le bilan hépatique et le dosage des hormones thyroïdiennes étaient normaux. La patiente était mise en condition, sous oxygénothérapie. L’insulinothérapie et l’antibiothérapie probabiliste à base d’amoxicilline acide clavulanique étaient instaurées. La radiographie thoracique et la tomodensitométrie thoracique avec des coupes passant par le foie et les surrénales avec une injection de produits de contraste étaient respectivement réalisées (Figure 1, Figure 2). La bronchoscopie souple objectivait une inflammation diffuse des bronches à la limite du 2ème degré sans autres anomalies visibles. La biopsie transbronchique à travers la lobaire moyenne était réalisée. L’échographie cervicale, abdomino-pelvienne et mammaire ainsi qu’une mammographie étaient sans particularité.

## Discussion

L’analyse du liquide d’aspiration bronchique objectivait la présence de cellules carcinomateuses. L’étude anatomopathologique d’un fragment biopsique provenant d’un parenchyme pulmonaire objectivait une prolifération tumorale, maligne, infiltrante, disposée en papilles et en tubes. Les cellules comportent des noyaux atypiques, irréguliers en faveur d’un adénocarcinome bien différencié de type papillaire et infiltrant du poumon. Une étude immunohistochimique montrait que les cellules tumorales exprimaient l’anti-TTF-1. Elles n’exprimaient pas la cytokératine 20. L’examen clinique, l’imagerie thoracique, thoraco-abdominale et mammaire et l’histologie de la biopsie transbronchique permettaient de retenir le diagnostic d’adénocarcinome bien différencié et infiltrant, d’architecture papillaire, primitif. Nous déplorons que l’évolution fût marquée, un mois après, par le décès de la patiente dans un tableau d’insuffisance respiratoire chronique. L’adénocarcinome est la forme histologique de cancer broncho-pulmonaire la plus fréquente représentant 30 à 35% de ces cancers [1]. Il est souvent lié au tabagisme [2]. Cependant, parmi les personnes qui n’ont jamais fumé, l’adénocarcinome est la forme la plus fréquente de cancer du poumon [3]. Les signes cliniques respiratoires ne sont pas spécifiques de la maladie et non plus les signes radiologiques [1]. L’aspect radiologique devant le syndrome interstitiel orienterait plus en faveur d’une atteinte pulmonaire d’une hémopathie maligne ou d’un poumon éosinophilique, vu le contexte clinique et radio-biologique [4, 5]. Cependant, il faut toujours penser à d’autres causes, comme une lymphangite carcinomateuse, une métastase d’un néo extra ou intra-thoracique, notamment d’un adénocarcinome, quoique ce dernier soit rare [6]. L’adénocarcinome pulmonaire a une localisation parenchymateuse périphérique, du fait qu’il naît de l’épithélium alvéolaire [1], ce qui pourrait expliquer cet aspect radiologique. Toute opacité de taille variable peut avoir plusieurs origines: bénigne, maligne primitive ou secondaire [6]. Les contours réguliers et nets orientent généralement vers l’étiologie bénigne quoique les cancers secondaires aient des limites bien nettes. Les cancers primitifs pourraient avoir également les mêmes aspects. Les nodules calcifiés ne sont pas toujours d’origine séquellaire ou bénigne mais aussi maligne. Il témoigne souvent des métastases osseuses comme cause maligne. Cependant, le carcinome broncho-génique, notamment l’adénocarcinome, ne peut être écarté [1, 6, 7]. La bronchoscopie est un examen systématique devant toute suspicion d’un carcinome broncho-génique. Les biopsies bronchiques et/ou les biopsies transbronchiques peuvent apporter le diagnostic. Devant l’atteinte parenchymateuse diffuse, outre le levage broncho-alvéolaire, la biopsie transbronchique est la clé du diagnostic [1, 6, 8]. C’est le moyen qui a permis d’obtenir le diagnostic étiologique dans notre cas. Le diagnostic d’adénocarcinome pulmonaire est difficile à retenir, d’où la nécessité de l’étude immunohistochimique, voire de l’étude moléculaire [9, 10]. Le pronostic du cancer broncho-pulmonaire reste sombre, comme en témoigne aussi le cas de notre patiente [11].

## Conclusion

L’adénocarcinome pulmonaire est difficile à évoquer d’emblée devant les données anamnestiques et même radiologiques. Cependant, il ne doit plus être ignoré devant toute image radiologique suspecte d’un cancer broncho génique, notamment l’adénocarcinome. Seule l’histologie aide à la confirmation diagnostique et l’étude immunohistochimique permet de distinguer au moins le caractère primitif ou secondaire. Le pronostic reste sombre dans ce type de cancer.
